# New Hydrogel Network Based on Alginate and a Spiroacetal Copolymer

**DOI:** 10.3390/gels7040241

**Published:** 2021-11-27

**Authors:** Alina Elena Sandu, Loredana Elena Nita, Aurica P. Chiriac, Nita Tudorachi, Alina Gabriela Rusu, Daniela Pamfil

**Affiliations:** “PetruPoni” Institute of Macromolecular Chemistry, Grigore Ghica Voda Alley 41-A, RO-700487 Iasi, Romania; sandu.alina@icmpp.ro (A.E.S.); achiriac@icmpp.ro (A.P.C.); ntudor@icmpp.ro (N.T.); rusu.alina@icmpp.ro (A.G.R.); dpamfil@icmpp.ro (D.P.)

**Keywords:** hybrid gel network, natural/synthetic polymer conjugate, alginate, poly(itaconic anhydride-co-3,9-divinyl-2,4,8,10-tetra-oxa-spiro[5.5]undecane), bio-applications

## Abstract

This study reports a strategy for developing a biohybrid complex based on a natural/synthetic polymer conjugate as a gel-type structure. Coupling synthetic polymers with natural compounds represents an important approach to generating gels with superior properties and with potential for biomedical applications. The study presents the preparation of hybrid gels with tunable characteristics by using a spiroacetal polymer and alginate as co-partners in different ratios. The new network formation was tested, and the structure was confirmed by FTIR and SEM techniques. The physical properties of the new gels, namely their thermal stability and swelling behavior, were investigated. The study showed that the increase in alginate content caused a smooth increase in thermal stability due to the additional crosslinking bridges that appeared. Moreover, increasing the content of the synthetic polymer in the structure of the gel network ensures a slower release of carvacrol, the encapsulated bioactive compound.

## 1. Introduction

Hydrogels are three-dimensional, cross-linked networks of polymers and their individual physical properties are of particular interest for use in drug delivery applications, including in pharmaceutical patches or Transdermal Therapeutical Systems (TTS). Some of the most important characteristics of the hydrogels refer to their soft consistency and elasticity, as well as compatibility to body tissues [1,2]. These properties endorse the hydrogels as very attractive structures for biomaterials uses [1,3,4,5,6]. Hydrogels based on combinations between natural and synthetic polymers offer significant advantages, e.g., tunable mechanical properties, increased water content, enhanced biocompatibility and appropriateness to body tissue, and possibility of attaching chemical clues for further superior interfacial interactions. The use of polysaccharides as a base for the three–dimensional network structure preparation—recommended due to their properties such as biocompatibility, obtainment from renewable sources, and possibilities of “green” procedures for their modifying—is of major interest [1,7]. Among these compounds, the alginate has an important place. Alginic acid sodium salt (Alg–Na) is a naturally occurring biopolymer, biodegradable, biocompatible, and non-inflammatory, successfully used in medical applications as a carrier for drug delivery [8,9,10]. In the form of physically and chemically cross-linked systems, the alginate is an attractive starting material for the construction of hydrogels with desired morphology, stiffness, and bioactivity. However, the short residence of alginate time, due to a fast degradation process and poor mechanical characteristics, strongly limit the possibility of broadening its range of biomedical applications. Several chemical transformations of native alginate have been designed to provide mechanically and chemically robust materials and expand its range of application [8,11,12,13]. Moreover, the combination of alginate with synthetic polymers for obtaining hydrogels is of interest because in this way, the resistance and reproducibility of the materials increase. There is a series of studies [14,15,16,17] concerning the structures with synergistic properties resulting from combining synthetic polymers with polysaccharides [18]. Our group reported the preparation of poly(itaconic anhydride-co-3, 9-divinyl-2,4,8,10-tetraoxaspiro (5.5) undecane) (PITAU), a synthetic copolymer with pendant functional groups. PITAU presents specific properties, such as the possibility to create networks, biodegradability and biocompatibility, binding properties, amphiphilicity, thermal stability, and also sensitivity to pH and temperature [19,20]. The versatility and untapped potential of these polymeric systems make them promising agents for pharmaceutical delivery systems or support for bioactive compounds, among other biomedical applications. Because of these special characteristics of the PITAU copolymers, and taking into account our previous studies [19,20], the possibility of grafting PITAU onto alginate, to obtain biocompatible gels with improved properties it was investigated in the present work. This study presents the preparation of bioconjugated gels based on alginate and poly(itaconic anhydride-co-3,9-divinyl-2,4,8,10-tetraoxaspiro[5.5]undecane). The new prepared structures were characterized from structural, morphological, and thermal behavior points of view.

## 2. Results and Discussion

**Figure 1:**
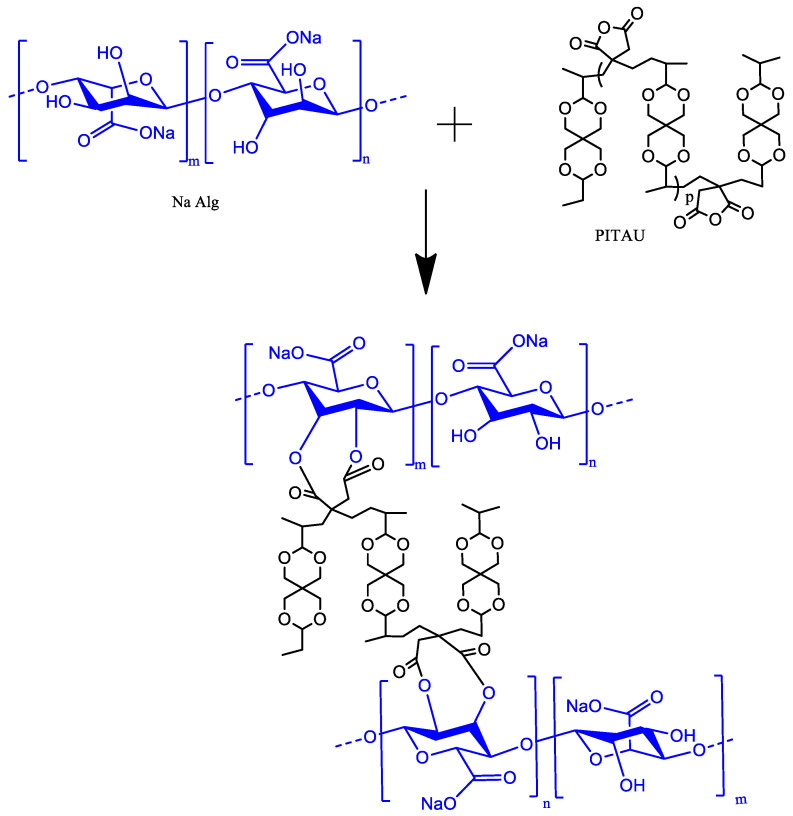
Schematized PITAU–alginate network.

The proposed illustration structure of the new synthesized gel based on alginate and PITAU is presented in Figure 1.

### 2.1. FTIR Spectra

**Figure 2:**
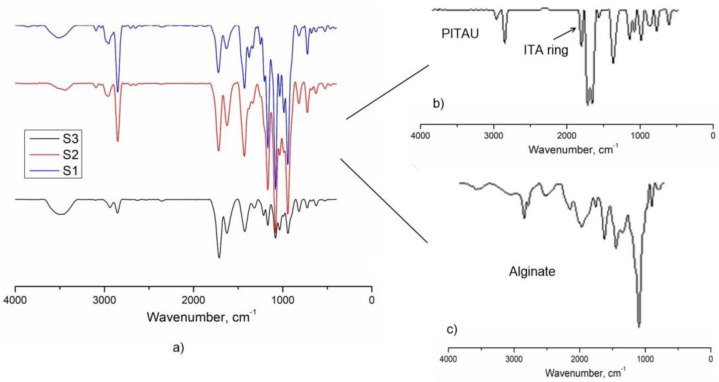
FTIR spectra of the (**a**) S1, S2, and S3 samples; (**b**) PITAU; (**c**) alginate.

The FTIR spectra of the gel samples are presented in Figure 2. The chemical composition of PITAU–Alg gels was confirmed by FTIR spectroscopy. The characteristic peaks of the PITAU copolymer appeared at (1) 1780 cm^−1^ and 1856 cm^−1^ corresponding to C=O symmetric and asymmetric stretching of the five–member anhydride unit; (2) 1660 cm^−1^ from C=C stretching; and (3) 1400 cm^−1^ for =CH_2_ in plane deformation, peaks evidenced as well by other authors [21]. The presence of a strong band around the 1000–1200 cm^−1^ region is attributed to ether C–O–C stretching from the spirochetal moieties. The presence of alginate is confirmed by characteristic alginate peaks registered at 2923 cm^−1^ and 2850 cm^−1^ that correspond to stretching vibrations of aliphatic C−H; at 1610 cm^−1^, corresponding to the carboxylic groups C–O–O as a result of the asymmetric stretch; and the symmetric stretching at 1419 cm^−1^. The band from 1024 cm^−1^ was attributed to the C–O stretching vibration, with contributions from C–C–H and C–O–H deformation data.

The disappearance of the peaks corresponding to the vibrations of the anhydride ring from 1782 cm^−1^ and 1862 cm^−1^ (Figure 2) confirm the covalent bonds between alginate and PITAU governed by OH groups of the alginate, which open the ITA anhydride ring.

### 2.2. SEM Studies

**Figure 3:**
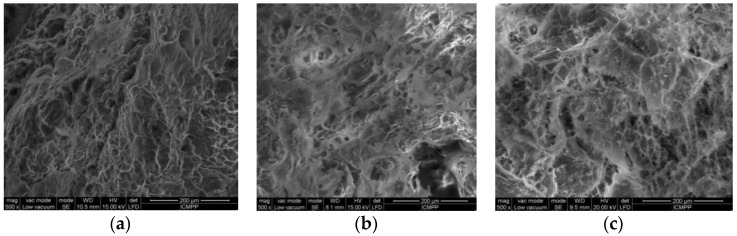
SEM microscopy of the freeze-dried gels: (**a**) S1, (**b**) S2, (**c**) S3.

The samples were analyzed using SEM microscopy to investigate the hydrogel microstructural architecture. The morphological analysis, illustrated in Figure 3, was performed in cross-section of the freeze–dried polymeric networks. According to the cross-sectional SEM pictures (Figure 3, S1–S3), the hydrogels present a continuous and porous configuration. As it is well known, the structural characteristics such as the porosity and topography governed by the chemical nature are very important in the performance of a gel. Moreover, a gel structure with a 3D network of interconnected channels and appropriate pores and pore size, allows a deeper penetration of liquids loaded with bioactive substances. The following images, representative of the studied gels, demonstrate the porous 3D architecture of the synthesized bioconjugate networks. SEM analyses confirm the important role of the amount of PITAU–Alg in generating the gel networks with superior properties. The pores of the gels have specific shapes and dimensions, in direct correlation with the PITAU–Alg ratio. Thus, the S3 sample, with higher amounts of alginate and smaller quantities of PITAU, leads to the formation of the irregular networks with larger pores. By increasing the PITAU amount (S2, S1), the gel networks are more ordered and present smaller pores. The morphological aspect of the bioconjugate matrices is correlated with the gel’s capacity for swelling (Figure 8) and the ability to further incorporate, transport, and release a therapeutic agent.

### 2.3. Thermal Degradation

**Figure 4:**
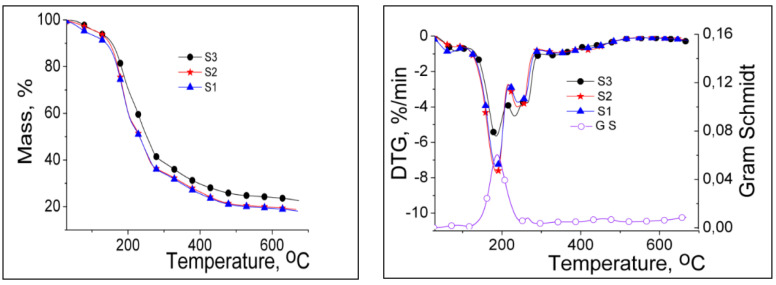
TG and DTG curves of PITAU–Alg gels.

Figure 4 illustrates the thermogravimetric (TG) and derivative thermogravimetric (DTG) curves of the studied samples obtained with different gravimetric ratios between PITAU and alginate co-partners, and Table 1 presents the main thermal parameters of the samples. TG and DTG curves of the samples show similar shapes with different mass losses in the five stages of degradation. In the first stage, the mass losses of 4.15–6.52% were determined on the moisture removal, including the water adsorbed on the surface. The mass losses of 29.28–40.79% were recorded in the second stage of degradation with T_onset_ between 145–147 °C, due to the cleavage of hydroxyl, carboxyl, and carbonyl groups from samples and release of H_2_O, CO_2_, aldehydes, alcohols, and ketones [22]. The increase in the temperature over 220 °C generates two consecutive thermal processes (III, IV), which occur with low decomposition rates (3.5–4.0%/min) depending on the gravimetric ratio between PITAU and Alg in gels. The last thermal process with T_onset_ above 300 °C led to the structural units’ decomposition of PITAU and the alginate glycosidic ring, with mass losses of about 16%, along with the release of CO_2_ and high–molecular weight aliphatic derivatives, and the obtaining of Na_2_CO_3_ due to Na–alginate dehydration and decarbonylation. At 650 °C, Na_2_CO_3_ partially decomposes into CO_2_ and Na_2_O as residue. The increase in the Na–alginate content in the PITAU–Alg samples determined a slight increase in the thermal stability, which was attributed to the additional crosslinking bridges that appear in the new system. This observation is also supported by T20 and T40 values (temperatures with mass losses of 20% and 40%, respectively). These values are 181 °C and 227 °C in the case of S3, 177 °C and 206 °C for S2, and 172 °C and 203 °C for S1. Therefore, the thermal stability series grows in the following order: S3 > S2 > S1.

**Figure 5:**
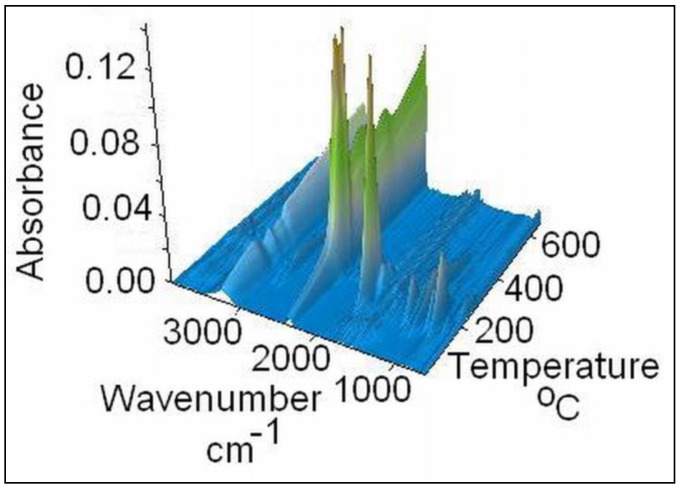
FTIR–3D spectrum of PITAU–Alg gel structure.

**Figure 6:**
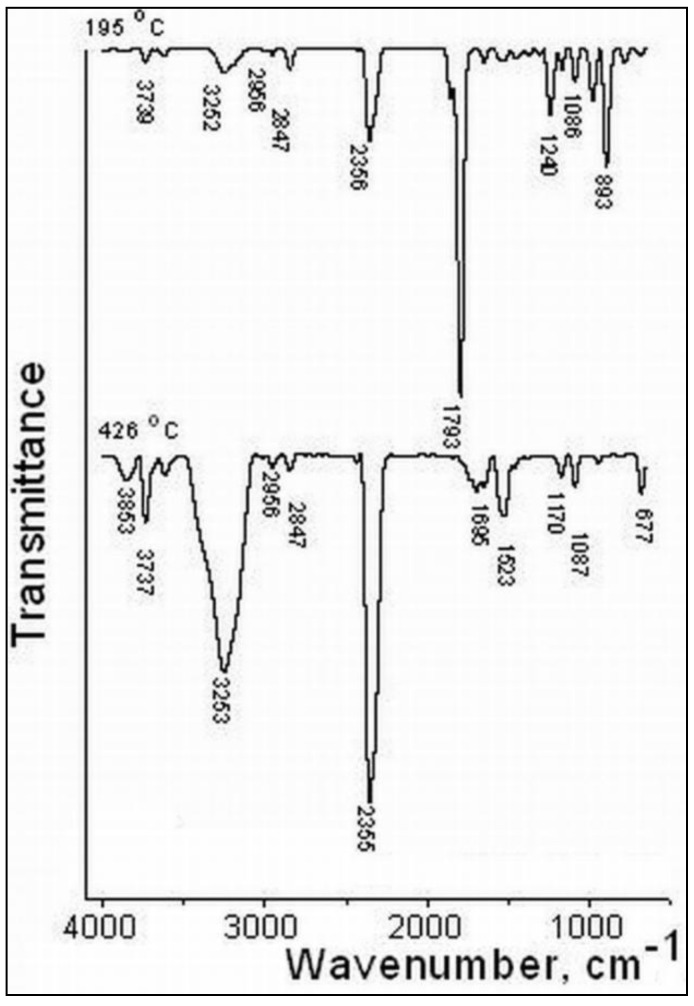
FTIR spectra of the evolved gases by thermal degradation of PITAU–Alg gel.

The gases released were also examined by FTIR and mass spectrometry techniques concomitantly with the thermal decomposition of the S3 sample on the TG/FTIR/MS system, in the temperature range of 30–650 °C. Their 3D FT–IR spectrum is illustrated in Figure 5, and it can be noticed that the release of major gases during thermal decomposition takes place between 150–400 °C, according to the Gram–Schmidt and DTG curves. The main gases were identified based on the IR spectra and MS signals available in the literature and spectral libraries of the NIST [23]. From the 3D FT–IR spectrum, we extracted 2D spectra corresponding to the released gases at 195 °C and 426 °C (Figure 6). The absorption band from 3253 cm^−1^ was assigned to the MCT detector (ice band) of the TGA–IR external module cooled with liquid nitrogen [24]. The major gaseous degradation products from the studied sample were water, carbon dioxide, carboxylic derivatives, alcohols, saturated and unsaturated aliphatic hydrocarbons, cycloalkanes, ketones, aldehydes, anhydrides, and ethers. Thus, the main absorption bands located at 3853–3619, 1375–1304, and 1240 cm^−1^ can be assigned to water vapors and alcohols that can appear at the thermal degradation of the secondary hydroxyl or ester groups from alginate and PITAU units. The absorption bands from 3062–2847, 1523, 1462–1454, and 977–893 cm^−1^ are assigned to the vibration of CH, CH_2_, and CH_3_ groups located in the chemical structure of the saturated and unsaturated aliphatic hydrocarbons, cycloalkanes, and aldehydes. The higher signal present at 2355 cm^−1^ and the small signal at 677 cm^−1^ are attributed to carbon dioxide. The absorption bands between 1695 and 1648 cm^−1^ are assigned to the asymmetric carbonyl groups in acids, aldehydes, and ketones, and those from 1854–1793 and 1170–1086 cm^−1^ (νC=O vibrations) in ethers, anhydrides, and unsaturated aldehydes. These data are in agreement with the chemical structure of PITAU–Alg gels and correspond with gases that may result from their thermal degradation.

**Figure 7:**
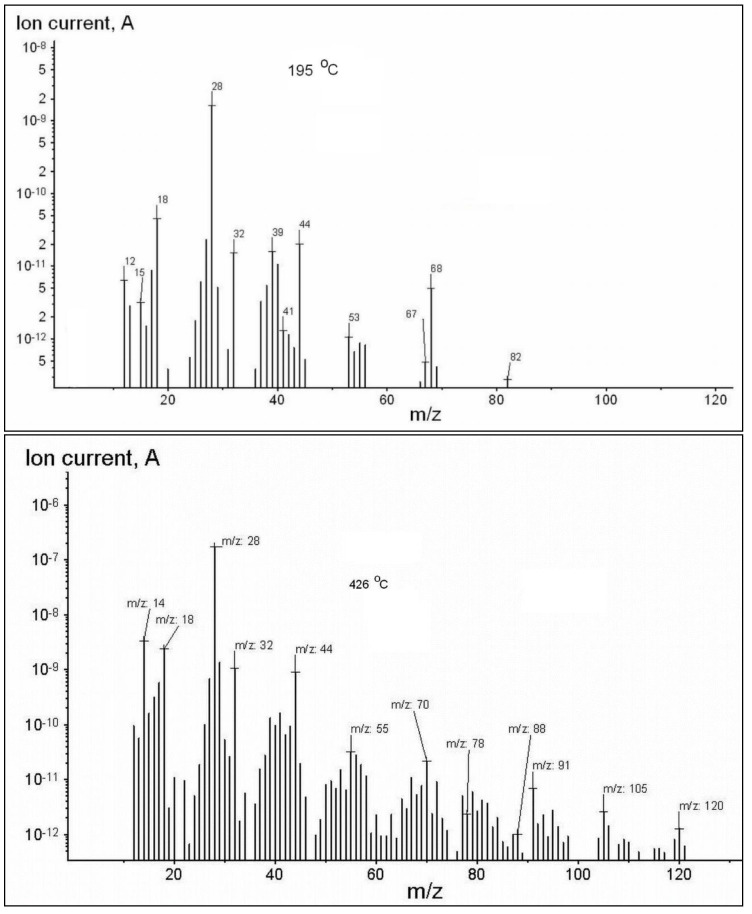
MS spectra of the evolved gases by thermal degradation of PITAU–Alg gel structures.

The data obtained from FTIR analysis of the evolved gases were also confirmed by MS spectrometry, and corresponding *m*/*z* signals are presented in Figure 6. With the increase in the temperature over 300 °C, a higher abundance of the gases developed, and the *m*/*z* ratio reached up to 120 for some high–molecular weight products. This confirms that up to 200 °C, there is a tearing of the thermal labile chemical bonds belonging to the functional groups (hydroxyl, carbonyl, carboxyl, and C–O links) from the structural units, and over 300 °C of the hydrocarbon bonds [25]. The main ionic fragments shown in Figure 7 are assigned as follows: HO^+^ (*m*/*z* = 17), H_2_O+ (*m*/*z* = 18), CO_2_^+^ (*m*/*z* = 44). The ionic fragments of some saturated and unsaturated aliphatic derivatives are assigned as follows: CH_2_^+^ (*m*/*z* = 14), CH_3_^+^ (*m*/*z* = 15), ethane C_2_H_6_^+^ (*m*/*z* =30), cyclopropane C_3_H_6_^+^ (*m*/*z* = 42), propane C_3_H_8_^+^ (*m*/*z* = 44), cyclobutene C_4_H_6_^+^ (*m*/*z* = 54), cyclobutene, 2–butene C_4_H_8_^+^ (*m*/*z* = 56), butane C_4_H_10_^+^ (*m*/*z* = 58), cyclohexane C_6_H_12_^+^ (*m*/*z* = 84), carbonyl derivatives such as formaldehyde CH_2_O^+^ (*m*/*z* = 30), acetaldehyde C_2_H_4_O^+^ (*m*/*z* = 44), acetone C_3_H_6_O^+^ (*m*/*z* = 58), 2–cyclopenten–1–one C_5_H_6_O^+^ (*m*/*z* = 82), itaconic anhydride C_5_H_4_O_3_ (*m*/*z* = 112), alcohols as methanol CH_3_OH+ (*m*/*z* = 32), ethanol C_2_H_5_OH^+^ (*m*/*z* = 46), propanol C_3_H_8_O^+^ (*m*/*z* = 60), acids as formic acid CH_2_O_2_^+^ (*m*/*z* = 46), acetic acid C_2_H_4_O_2_^+^ (*m*/*z* = 60), propionic acid C_3_H_6_O_2_^+^ (*m*/*z* = 74), vinyl ethyl ether C_4_H_8_O^+^ (*m*/*z* = 72), diethyl ether C_4_H_10_O^+^ (*m*/*z* = 74).

### 2.4. Swelling Study

**Figure 8:**
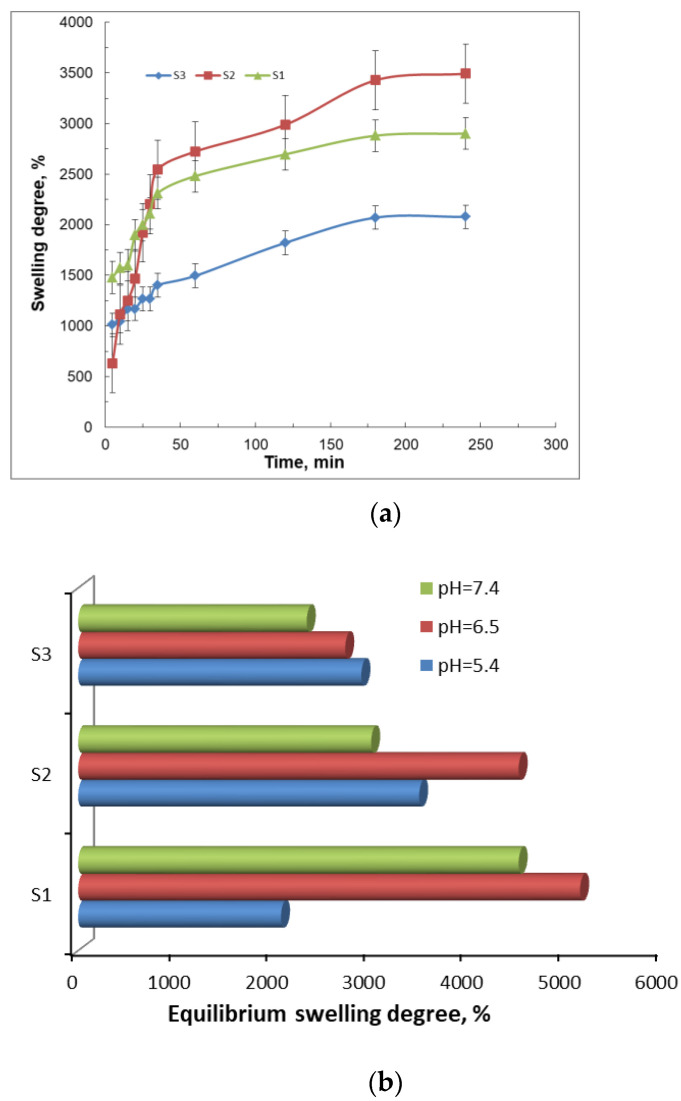
The swelling behavior of the gels as a function of time (**a**) and equilibrium swelling degree at different pH values (**b**).

One of the most important properties of the gels with pharmaceutical applicability is their capacity to swell when they come in contact with thermodynamically compatible solvents. In this case, the solvent molecules penetrate the polymeric network and determine the expanding of pores, which allow the incorporation of the drug or of other solvent molecules. As is well known, the swelling process is a consequence of the polymer–fluid interactive forces, which increase with the hydrophilic character of the macromolecules. The swelling degree of the studied gel samples as a function of time is illustrated in Figure 8. All the samples show a burst increase in swelling at the early stage, e.g., within the first 10 min. Then, the process is slowly continued up to 300 min.

The maximum degree of swelling corresponds to the network with the average amount of PITAU and alginate (S2), which is justified by a network structure with regulated pores (Figure 3b), due to PITAU copolymer presence, but at the same time owing to the alginate chains with relative mobility, which facilitates swelling. On the other hand, S1 samples have more possibilities for intense intramolecular bonds between alginate and PITAU governed by OH groups of the alginate, which open the ITA anhydride ring. The samples are pH–sensitive (Figure 8b), with the maximum swelling capacity registered at pH = 6.5.

The minimum swelling capacity is recorded for the sample with the minimum amount of PITAU and maximum amount of alginate (S3). In this case, the compact structure of the system induced a reduced swelling capacity. Subsequently, with the addition of synthetic polymer and the generation of an intermolecular network, the penetration of solvent molecules is easier, and consequently, the swelling capacity increased.

### 2.5. Release Study

**Figure 9:**
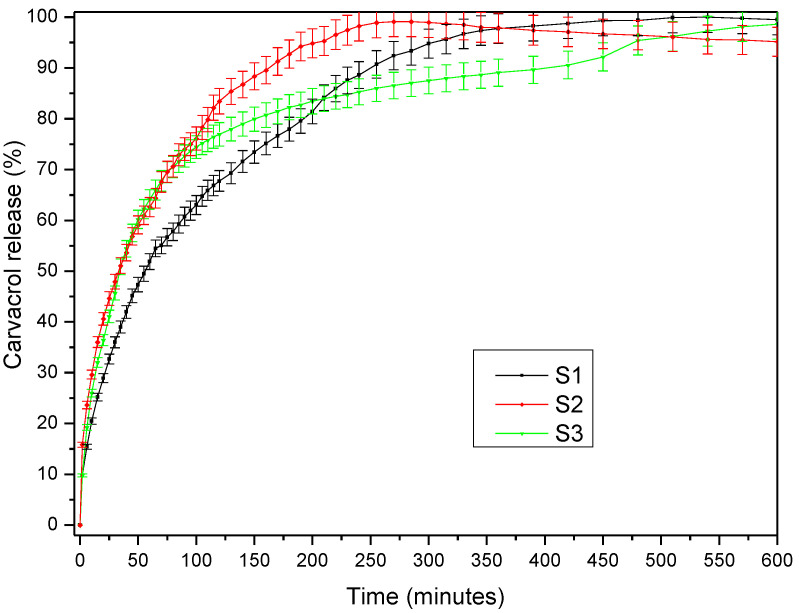
The release profile of carvacrol from PITAU–Alg hydrogel network.

The capacity of the PITAU–Alg network structure as a matrix was tested by the encapsulation and release of carvacrol. The carvacrol release profile presented in Figure 9 illustrates a burst effect highlighted in the first minutes of the samples’ immersion in medium, while the equilibrium was reached after 4–11 h, depending on the PITAU–Alg hydrogel composition. Thus, the maximum amount of the drug released from the S2 hydrogel was reached after only 250 min, followed by the S1 hydrogel composition, with a more prolonged release time and a plateau reached after 540 min. The S3 hydrogel variant proceeds a fast release of bioactive compounds at the beginning, but then an extended release up to 720 min was observed. These observations correlate with the swelling study that attests a smaller degree of swelling in the case of the S3 sample and a higher degree for the S2 sample.

The drug release kinetic parameters presented in Table 2 were calculated using the semi–empirical equation proposed by Korsmeyer and Peppas [26]. In the table above, the value of *n* = 0.4931 obtained for S1 indicates a Fickian diffusion mechanism of the drug from the sample, while the value of *n* = 0.5581 obtained for S3, which is between 0.5 < *n* < 1, indicates an anomalous non–Fickian release behavior. The highest value of release rate constant k was obtained for S1, indicating the most accelerated drug release behavior, as observed in Figure 9, due to the chain disentanglement. These observations are also correlated with SEM images (Figure 3) which display a more ordered gel network with smaller pores in the S1 sample, in accord with the Fickian mechanism of release.

## 3. Materials and Methods

### 3.1. Materials

All reagents were of analytical purity and were used without further purification: alginic sodium salt (Alg) from brown algae was supplied by Acros Organics from Belgium, 3,9-divinyl-2,4,8,10-tetraoxaspiro[5.5] undecane (U) (purity 98%, Sigma-Aldrich, Hamburg, Germany), itaconic anhydride (ITA) (purity 98%, Aldrich), and 2,2′-Azobis (2-methylpropionitrile) (AIBN) (purity 98%, Sigma-Aldrich).

The solvents that we used 1,4 dioxane (D) (purity ≥ 99.0%) and diethyl ether (for precipitation)—were purchased from Sigma–Aldrich. The water used in the experiments was purified using an Ultra Clear TWF UV System.

### 3.2. Copolymer Synthesis

The PITAU preparation was described in detail before [20]. In brief, PITAU copolymers were synthesized through a radical process of polymerization with a total monomer concentration of about 20% (ratio between itaconic acid (ITA)/3, 9-divinyl-2,4,8,10-tetraoxaspiro (5.5) undecane (U) = 1.5/1), using AIBN as an initiator (0.9%) and 1,4-dioxane as a solvent. The reaction was conducted under a nitrogen atmosphere, in a constant temperature bath at 75 °C, with a stirring rate of 250 rpm, for about 17 h. The reaction mixture was further added dropwise into diethyl ether when the copolymer precipitated. The copolymer was further washed several times with diethyl ether and dried in a vacuum oven at room temperature with a 600 mm HG vacuum for 24 h.

### 3.3. Bioconjugate Samples Preparation

Three variants of bioconjugate structures based on PITAU and Alg were prepared as presented in Table 3. Precise amounts of copolymer (in dioxane solution) were mixed with specific amounts of alginate aqueous solution in order to have the following gravimetric ratios: PITAU:Alg = 3:1; 2:1, and 1.5:1. The gels formed rapidly within 20 min after mixing, and were left to mature for 24 h. Then, the gels were freeze–dried to remove the solvents and use the systems for further investigations.

### 3.4. Bioactive Compound Preparation

The carvacrol (Sigma Aldrich, Hamburg, Germany) was used as an antimicrobial compound. The ratio between the polymer and carvacrol was 2:1. The freeze–dried gels were immersed into a carvacrol solution and immersion was realized by diffusion mechanism. All the liquid was adsorbed into a polymer matrix.

### 3.5. Characterization of PITAU–Alg Gels

#### 3.5.1. FT–IR Spectra

FT–IR spectra of the synthesized samples (S1, S2, S3) were recorded on a Vertex Bruker Spectrometer (Germany) in an absorption mode ranging from 400 to 4000 cm^−1^. The samples were grounded with potassium bromide (KBr) powder and compressed into a disc for analysis. The spectra were generated at 4 cm^−1^ resolution with an average of 64 scans.

#### 3.5.2. SEM Studies

The microstructural architecture of the samples was analyzed using SEM microscopy. The freeze-dried samples were examined by using a scanning electron microscope (Quanta 200). According to the cross–sectional SEM pictures, the hydrogels displayed a continuous and porous configuration acquired after freeze-drying.

#### 3.5.3. Thermal Analysis

Thermogravimetric behavior of the samples was studied in dynamic conditions using a STA 449 F1 Jupiter apparatus (Netzsch, Germany) in a nitrogen atmosphere with a 10 °C min^−1^ heating rate, in the temperature range of 30–650 °C. Samples of 10–15 mg were placed in Al_2_O_3_ crucibles, and Al_2_O_3_ was used as reference material. The gases appearing by the thermal degradation of the samples were analyzed using an online connected spectrophotometer FT–IR (Vertex 70) equipped with an external module TGA–IR and Aëolos QMS 403C mass spectrometer. The acquisition of FT–IR spectra in 3D was executed with OPUS 6.5 software, with spectra recorded at 600–4000 cm^−1^ intervals, at a resolution of 4 cm^−1^. The QMS 403C spectrometer worked at 10–5 mbar vacuum and electron impact ionization energy of 70 eV. The data acquisition was achieved in the range *m*/*z* = 1–200 with a measurement time of 0.5 s for one channel, resulting in a time/cycle of 100 s.

#### 3.5.4. The Swelling Studies

The swelling degree of the gel samples was determined in PBS (phosphate-buffered saline) (0.01 M at pH = 5.4) and at 25 °C room temperature. The amount of the adsorbed solution was gravimetrically determined: the swollen gels were regularly taken out from the swelling medium, soaked on filter paper surface, weighed, and placed after every weighing in the same swelling medium. The measurements were continued until a constant weight was reached for each studied sample. All swelling experiments were performed in triplicate for S1, S2, and S3 samples. The degree of swelling (SD) was calculated by the following equation:

$$SD,\;\%=\frac{M\left(t\right)-M_{0}}{M_{0}}\;\times \;100$$

where *M*(*t*) is the weight of the swollen particles at time *t*, and *M*_0_ is the weight of the sample before swelling (the weight of the dry sample).

The experiment was carried out in triplicate.

#### 3.5.5. The Release Study

The in vitro release studies of carvacrol were carried out in a 708–DS Dissolution Apparatus coupled with a Cary 60 UV–VIS spectrophotometer (from Agilent Technologies, Germany) in 100 mL medium (85 mL 7.4 pH PBS/15 mL ethanol) at 25 ± 0.5 °C with a rotation speed of 100 rpm. Aliquots of the medium withdrawn at predetermined time intervals were analyzed with a λ_max_ value of 273 nm. The carvacrol concentrations were calculated based on the calibration curves determined at the same wavelengths.

The experiment was carried out in triplicate.

## 4. Conclusions

New gel network systems based on PITAU and alginate were developed in order to observe their characteristics, and to predict their potential applications in the biomedical and pharmaceutical fields.

According to SEM microscopy, the synthesized gels presented a continuous and porous morphology. SEM analyses also confirmed the important role of the ratio between PITAU and Alg co-partners in generating the bioconjugated structures with improved crosslinked networks. The also study underlined the dependence of the swelling properties of the synthesized networks on the ratio between PITAU and Alg co-partners. We found a better swelling capacity for the S2 sample (PITAU_Alg_2_1), justified by the larger pores generated by the intermolecular physical bonds between the macromolecular chains of the system. The study also demonstrated that the increase in the alginate content in the PITAU–Alg structures determined a smooth growth of the thermal stability, due to the additional crosslinking bridges that occur with increasing the amount of alginate. Moreover, the increase in the synthetic polymer content in the gel network structure ensured a slower release of carvacrol, the encapsulated bioactive compound.

## Figures and Tables

**Table 1:** Thermal parameters.

Sample	Degradation Stage	T_onset_ °C	T_peak_ °C	W%	T_20_, °C	T_40_, °C	GS °C
S3	I	40	84	5.18	181	227	86
	II	145	185	29.28			190
	III	217	232	17.59			266
	IV	259	264	9.27			340
	V	309	325	16.13			460
	residue			22.55			
S2	I	42	86	4.15	177	206	
	II	147	193	40.79			
	III	224	244	10.39			
	IV	252	259	9.67			
	V	309	347	16.26			
	residue			18.74			
S1	I	34	71	6.52	172	203	
	II	144	189	39.56			
	III	224	243	11.28			
	IV	255	258	8.35			
	V	305	337	16.20			
	residue			18.09			

Heating rate—10 °C/min; T_onset_—the temperature at which the thermal degradation starts; T_peak_—the temperature at which the degradation rate is maximum; T_20_, T_40_,—the temperatures corresponding to 20% and 40% mass losses, respectively; T_GS_—the temperature at which the maximum amount of gases was released (determined from Gram–Schmidt curves using *Proteus* software); W—mass losses up to 680 °C.

**Table 2:** Carvacrol release kinetic parameters.

Sample Name	*n*	R*_n_*^2^	*k* (min^−*n*^)	R*_k_*^2^
S1	0.4931	0.9976	0.0668	0.9971
S2	0.4055	0.9969	0.1209	0.9976
S3	0.5581	0.9982	0.0683	0.9989

*n*—release exponent; *k*—release rate constant; R*_k_*^2^ and R*_n_*^2^—correlation coefficients corresponding to the slope obtained for determination of *n* and *k*, respectively.

**Table 3:** Codification and chemical composition of the studied samples.

Tested Samples	Gel Code	PITAU (g)	Alginate (g)
S1	PITAU_Alg_3_1	6.50	2.15
S2	PITAU_Alg_2_1	5.00	2.50
S3	PITAU_Alg_1.5_1	4.00	2.65

The recipe was calculated to obtain 100 mL gel.

## References

[1] Nita L.E., Chiriac A.P., Bercea M., Ghilan A., Rusu A.G., Dumitriu R.P., Mititelu–Tartau L. (2019). Multifunctional hybrid 3D network based on hyaluronic acid and a copolymer containing pendant spiroacetal moieties. Int. J. Biol. Macromol..

[2] Fisher O.Z., Khademhosseini A., Langer R., Peppas N.A. (2010). Bioinspired materials for controlling stem cell fate. Acc. Chem. Res..

[3] Fajardo A.R., Pereira A.G.B., Rubira A.F., Valente A.J.M., Muniza E.C., Matricardi P., Alhaique F., Coviello T. (2016). Stimuli responsive polysaccharide–based hydrogels. Polysaccharide Hydrogels Characterization and Biomedical Applications.

[4] Costa D., Valente A.J.M., Miguel M.G., Queiroz J. (2011). Gel network photodisruption: A new strategy for the codelivery of plasmid DNA and drugs. Langmuir.

[5] Dugan J.M., Gough J.E., Eichhorn S.J. (2013). Bacterial cellulose scaffolds and cellulose nanowhiskers for tissue engineering. Nanomedicine.

[6] Durst C.A., Cuchiara M.P., Mansfield E.G., West J.L., Grande–Allen K.J. (2011). Flexural characterization of cell encapsulated PEGDA hydrogels with applications for tissue engineered heart valves. Acta Biomater..

[7] Van Vlierberghe S., Dubruel P., Schacht E. (2011). Biopolymerbased hydrogels as scaffolds for tissue engineering applications: A review. Biomacromolecules.

[8] Diaconu A., Nita L.E., Chiriac A.P., Mititelu–Tartau L., Doroftei F., Vasile C., Pinteala M. Self-linked polymer gels [based on hyaluronic acid and poly(itaconic anhydride-co-3,9-divinyl-2,4,8,10-tetraoxaspiro[5.5]undecane)] as potential drug delivery networks. Proceedings of the 5th IEEE International Conference on E-Health and Bioengineering—EHB.

[9] Callesa J.A., Tártarac L.I., Lopez-Garcíad A., Dieboldd Y., Palmac S.D., Vallésa E.M. (2013). Novel bioadhesive hyaluronan–itaconic acid crosslinked films for ocular therapy. Int. J. Pharm..

[10] Hoarea T.R., Kohaneb D.S. (2008). Hydrogels in drug delivery: Progress and challenges. Polymer.

[11] Borzacchiello A., Mayol L., Ramires P.A., Bartolo C., Pastorello A., Ambrosio L., Milella E. (2007). Structural and rheological characterization of hyaluronic acid–based scaffolds for adipose tissue engineering. Biomaterials.

[12] Borzacchiello A., Mayol L., Schiavinato A., Ambrosio L. (2010). Effect of hyaluronic acid amide derivative on equine synovial fluid viscoelasticity. J. Biomed. Mater. Res. A.

[13] Maltese A., Bucalo C., Maugeri F., Borzacchiello A., Mayol L., Nicolais L., Ambrosio L. (2006). Novel polysaccharides based viscoelastic formulations for ophthalmic surgery: Rheological characterization. Biomaterials.

[14] Imrea B., Garcíab L., Pugliad D., Vilaplana F. (2019). Reactive compatibilization of plant polysaccharides and biobased polymers: Review on current strategies, expectations and reality. Carbohydr. Polym..

[15] Vasile C., Pamfil D., Stoleru E., Baican M. (2020). New Developments in Medical Applications of Hybrid Hydrogels Containing Natural Polymers. Molecules.

[16] Rippe M., Cosenza V., Auzely–Velty R. (2019). Design of Soft Nanocarriers Combining Hyaluronic Acid with Another Functional Polymer for Cancer Therapy and Other Biomedical Applications. Pharmaceutics.

[17] Hu Y., Hu S., Zhang S., Dong S., Hu J., Kang L., Yang X. (2021). A double-layer hydrogel based on alginate–carboxymethyl cellulose and synthetic polymer as sustained drug delivery system. Sci. Rep..

[18] Diaconu A., Nita L.E., Bercea M., Chiriac A.P., Rusu A.G., Rusu D. (2017). Hyaluronic acid gels with tunable properties by conjugating with a synthetic copolymer. Biochem. Eng. J..

[19] Chiriac A.P., Nita L.E., Diaconu A., Bercea M., Tudorachi N., Pamfil D., Mititelu-Tartau L. (2017). Hybrid gels by conjugation of hyaluronic acid with poly(itaconic anhydride-co-3,9-divinyl-2,4,8,10-tetraoxaspiro[5.5]undecane) copolymers. Int. J. Biol. Macromol..

[20] Diaconu A., Chiriac A.P., Nita L.E., Tudorachi N., Neamtu I., Vasile C., Pinteala M. (2015). Design and synthesis of a new polymer network containing pendant spiroacetal moieties. Des. Monomers Polym..

[21] Shang S., Huang S.J., Weiss R.A. (2009). Synthesis and characterization of itaconic anhydride and stearyl methacrylate copolymers. Polymer.

[22] Liua Y., Zhang C.J., Zhao J.C., Guo Y., Zhu P., Wang D.Y. (2016). Bio-based barium alginate film: Preparation, flame retardancy and thermal degradation behavior. Carbohydr. Polym..

[23] Search for Species Data by Chemical Name. http://webbook.nist.gov/chemistry/name–ser.html.

[24] TGA–IR User Manual, Bruker. https://www.bruker.com/en/products-and-solutions/infrared-and-raman/ft-ir-research-spectrometers/tg-ftir-thermogravimetric-analysis.html.

[25] Nita L.E., Chiriac A.P., Rusu A.G., Bercea M., Diaconu A., Tudorachi N. (2018). Interpenetrating polymer network systems based on poly(dimethylaminoethyl methacrylate) and a copolymer containing pendant spiroacetal moieties. Mater. Sci. Eng..

[26] Korsmeyer R.W., Lustig S.R., Peppas N.A. (1986). Solute and penetrant diffusion in swellable polymers. I. Mathematical modeling. J. Polym. Sci. Part B Polym. Phys..

